# Fusion of Video and Inertial Sensing for Deep Learning–Based Human Action Recognition

**DOI:** 10.3390/s19173680

**Published:** 2019-08-24

**Authors:** Haoran Wei, Roozbeh Jafari, Nasser Kehtarnavaz

**Affiliations:** 1 Department of Electrical and Computer Engineering, University of Texas at Dallas, Richardson, TX 75080, USA; 2 Center for Remote Health Technologies and System, Texas A&M University, College Station, TX 77843, USA

**Keywords:** fusion of video and inertial sensing for action recognition, deep learning-based action recognition, decision-level and feature-level fusion for action recognition

## Abstract

This paper presents the simultaneous utilization of video images and inertial signals that are captured at the same time via a video camera and a wearable inertial sensor within a fusion framework in order to achieve a more robust human action recognition compared to the situations when each sensing modality is used individually. The data captured by these sensors are turned into 3D video images and 2D inertial images that are then fed as inputs into a 3D convolutional neural network and a 2D convolutional neural network, respectively, for recognizing actions. Two types of fusion are considered—Decision-level fusion and feature-level fusion. Experiments are conducted using the publicly available dataset UTD-MHAD in which simultaneous video images and inertial signals are captured for a total of 27 actions. The results obtained indicate that both the decision-level and feature-level fusion approaches generate higher recognition accuracies compared to the approaches when each sensing modality is used individually. The highest accuracy of 95.6% is obtained for the decision-level fusion approach.

## 1. Introduction 

Human action recognition has been extensively studied in the literature and has already been incorporated into commercial products. There are a wide range of applications of human action recognition including human–machine interface, e.g., [1,2,3,4], intelligent surveillance, e.g., [5,6,7,8], content-based data retrieval, e.g., [9,10], and gaming, e.g., [11,12].

Vision and inertial sensing modalities have been used individually to achieve human action recognition, e.g., [13,14,15,16,17,18,19,20,21,22,23]. Furthermore, the use of deep learning models or deep neural networks have proven to be more effective than conventional approaches for human action recognition. For example, in [15], it was shown that deep learning networks utilizing video images performed better than the previous conventional approaches. More recently, in [16], a three-dimensional (3D) convolutional neural network (CNN) was used by considering video data volumes. Video cameras are cost-effective and widely available. On the other hand, they have limitations in terms of their limited field of view and sensitivity to lighting and illumination changes. Depth cameras have also been utilized for human action recognition, e.g., [2,11]. The use of these cameras has been limited to indoor environments as they rely on infrared light for obtaining depth images. 

Furthermore, wearable inertial sensors have been used to achieve human action recognition. Similarly, these sensors are cost-effective and widely available. The main advantage of these sensors include their wearability—Thus, they are not limited to a specific field of view. Often, 3-axis acceleration signals from their accelerometers and 3-axis angular velocity signals from their gyroscopes are used for conducting human action recognition, e.g., [17,18,19,20,21,22,23]. These sensors have also limitations in terms of not capturing a complete representation of actions. The use of multiple inertial sensors, though possible, introduces its own challenge in terms of intrusiveness to wear multiple sensors on the body for capturing a complete representation of actions. 

Basically, no sensing modality is perfect: no sensing modality provides the entire information associated with various actions. In previous works on fusing two sensing modalities [24,25,26,27,28,29,30], it was shown that the fusion of the sensing modalities of depth camera and inertial sensor generated more robust human action recognition compared to when using each sensing modality individually. In this paper, the fusion of the sensing modalities of video camera and inertial sensor is examined to achieve a more robust human action recognition in terms of recognition errors compared to the situations when each sensing modality is used individually. Furthermore, this paper considers the use of deep learning models for this fusion. A 3D convolutional neural network is used for video data captured by a video camera and a 2D (two dimensional) convolutional neural network is used for inertial signals captured by a wearable inertial sensor. Both feature-level and decision-level fusion are examined. This is the first time a simultaneous utilization of video images and inertial signals are considered to achieve human action recognition. No other works are reported in the literature in which both video images and inertial signals are captured and processed at the same time to conduct human action recognition. 

The rest of the paper is organized as follows: Section 2 covers the dataset used. The architectures of the deep learning models used are presented in Section 3. The experimental results are reported in Section 4, followed by their discussion in Section 5. Finally, the paper is concluded in Section 6.

## 2. UTD-MHAD Dataset 

This section gives a brief description of the dataset utilized and the way the video images and inertial signals are fed into the deep learning models described in Section 3. The dataset used is a public domain dataset called the University of Texas at Dallas Multimodal Human Action Dataset (UTD-MHAD). Interested readers are referred to [31] for the details of this dataset. 

The UTD-MHAD dataset was collected by a Kinect camera and a wearable inertial sensor at the same time. The dataset consists of video images with a resolution of 640 × 480 and depth images with a resolution of 320 × 240 at 30 frames per second captured by the Kinect camera. It also consists of 3-axis acceleration and 3-axis angular velocity signals captured at the same time at a sampling rate of 50Hz by the inertial sensor. The details of the inertial sensor are provided in [32]. 27 different actions are included in this dataset. As listed in Table 1, actions numbered 1 through 21 are hands type movements. For these actions, the inertial sensor was worn on the right wrist. Actions numbered 22 to 27 are leg type movements. For these actions, the inertial sensor was worn on the right thigh.

Each of the actions were performed by eight subjects (four females and four males), and each action was repeated four times. Hence, there are a total of 864 action clips, with three of the data corrupted action clips not included. Each action clip consists of videos, depth videos, skeleton joint positions, and inertial sensor signals. In this work, the videos and the inertial signals are used.

**Figure 1:**
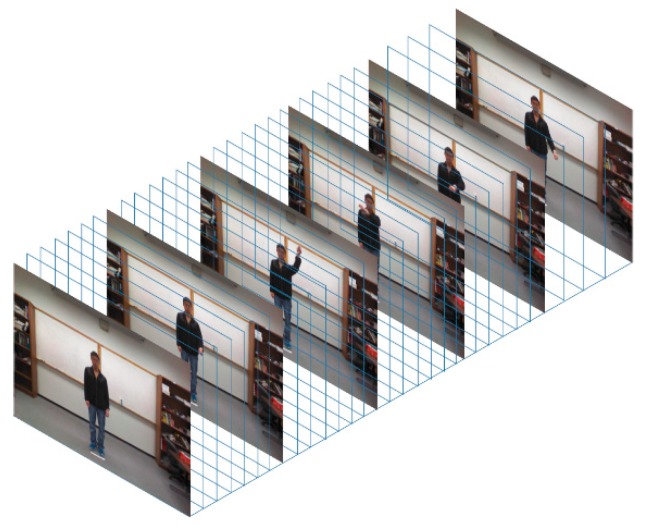
Example video volume as input to the 3D convolution neural network.

For the video data, since different actions have different video clip durations, 32 frames across each video clip are selected to form a consistent 3D input to the 3D convolutional neural network discussed in the next section. To gain computational efficiency, each frame is resized to 320 × 240 pixels. As a result, each action clip has the same spatial and temporal size forming a 320 × 240 × 32 3D video data volume. An example 3D video data volume formed in this manner is shown in Figure 1.

**Figure 2:**
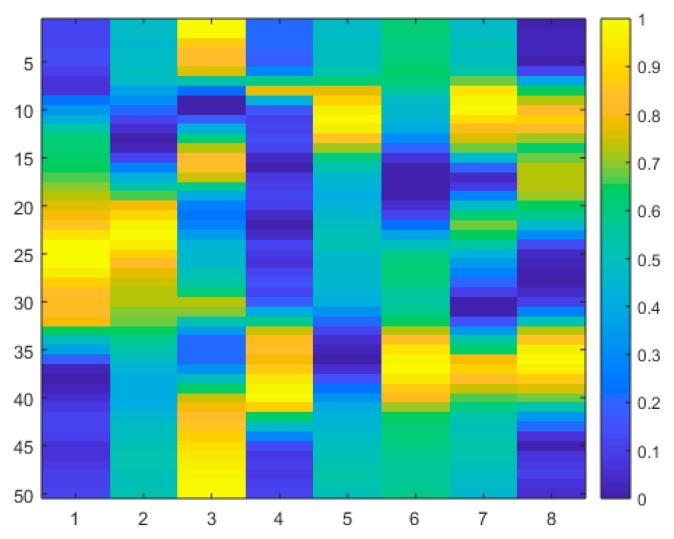
Example inertial signals image as input to the 2D convolution neural network.

For the inertial data, an eight-column input image is formed by combining the 3-axis acceleration signals, the 3-axis angular velocity signals, the overall acceleration signal, and the overall angular velocity signal at a sampling frequency of 50Hz. Thus, input images to the 2D convolutional neural network discussed in the next section are of size 8 × 50. An example inertial signals input image is shown in Figure 2.

## 3. Deep Learning Models 

**Figure 3:**
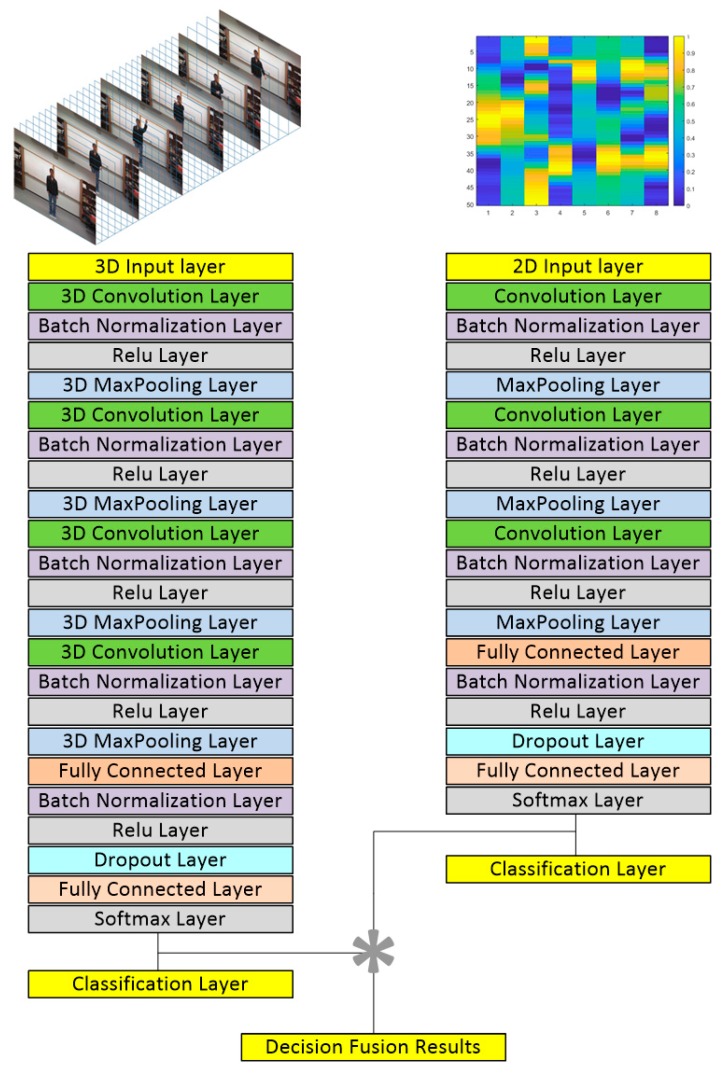
Network architecture used for the decision-level fusion of video and inertial sensing modalities.

A 3D convolutional neural network is used to learn the video data for the 27 actions of the UTD-MHAD dataset. The architecture of this model is a simplified version of the model discussed in [16] and is shown on the left side of Figure 3. More specifically, the input is a 320 × 240 × 32 3D video volume. The first 3D convolutional layers have 16 filters, and their outputs are passed through 3D max pooling layers. The second 3D convolutional layers convolve the outputs of the pooling layers with 32 filters and pass them to another set of 3D max pooling layers. The third and fourth 3D convolutional layers have 64 and 128 filters, respectively, passing outputs to 3D max pooling layers. All of the 3D convolution filters are of size 3 × 3 × 3 with stride 1 × 1 × 1. All the 3D pooling layers are of size 2 × 2 × 2 with stride 2 × 2 × 2. Batch normalization layers and Rectified Linear Unit (ReLU) activation are used at each 3D convolutional layer. The output of the last 3D pooling layer is flattened and passed to a fully connected layer with 256 units based on the ReLU activation. The output of the fully connected layer is passed to a 50% dropout layer and then connected to a final fully connected layer using the softmax cost function, producing scores for the output classes denoting the 27 actions. The stochastic gradient descent with momentum (SGDM) optimization algorithm is used to train the networks. Table 2 provides the architecture and training parameters associated with the 3D convolutional neural network used. 

For the inertial data, a 2D CNN is used. As inertial sensor signals are time-series signals, the 2D CNN model developed in [33] for speech processing is used here. This model is shown on the right side of Figure 3. It consists of 3 convolutional layers with 16, 32, and 64 filters, respectively, all having a size of 3 × 3. The 2D max pooling layers merge the filtering operations of the convolutional layers. The max pooling size used here is 2 × 2. At the last stage, there are two fully connected layers with 256 and 27 units, respectively. The scores of each class or action are then computed from the output layer using the softmax cost function. The ReLU activation is used at each convolutional layer and the first fully connected layer. The decision-level fusion is performed by multiplying the scores of the two sensing modalities and the class or action with the highest score is taken to be the recognized action. Table 3 provides the architecture and training parameters associated with the 2D convolutional neural network used.

A brief overview of the layers is described next. Interested readers are referred to many references that are available on deep neural networks, for example [34,35,36]. Three-dimensional convolutions are similar to 2D convolutions, but instead of using 2D filters, 3D filters are used. The 3D max pooling layers perform the down sampling operation by dividing 3D inputs to cuboidal pooling regions, and then by computing the maximum value of each such region. The ReLU activation function is a piecewise linear function, whose output is the same as the input when the input is positive and zero otherwise. Compared to other activation functions, ReLU exhibits more sensitivity to the input sum activation and avoids saturation and has become the default activation function in many computer vision and speech recognition applications. To speed up the training process and reduce sensitivity to network initialization, batch normalization layers are used between convolution layers and the ReLU layers. The batch normalization layers normalize each input layer across a batch by subtracting the batch mean and dividing by the batch standard deviation. Outputs of batch normalization layers maintain a mean value close to 0 and a standard deviation close to 1, allowing independency of the layers. Dropout layers reduce overfitting and improve the generalization capability of the networks by randomly dropping out nodes during training. The softmax cost function is used to transform a vector of input numbers to a probability distribution. After applying softmax, each output component lies in the interval between 0 and 1 with the sum adding up to 1.

**Figure 4:**
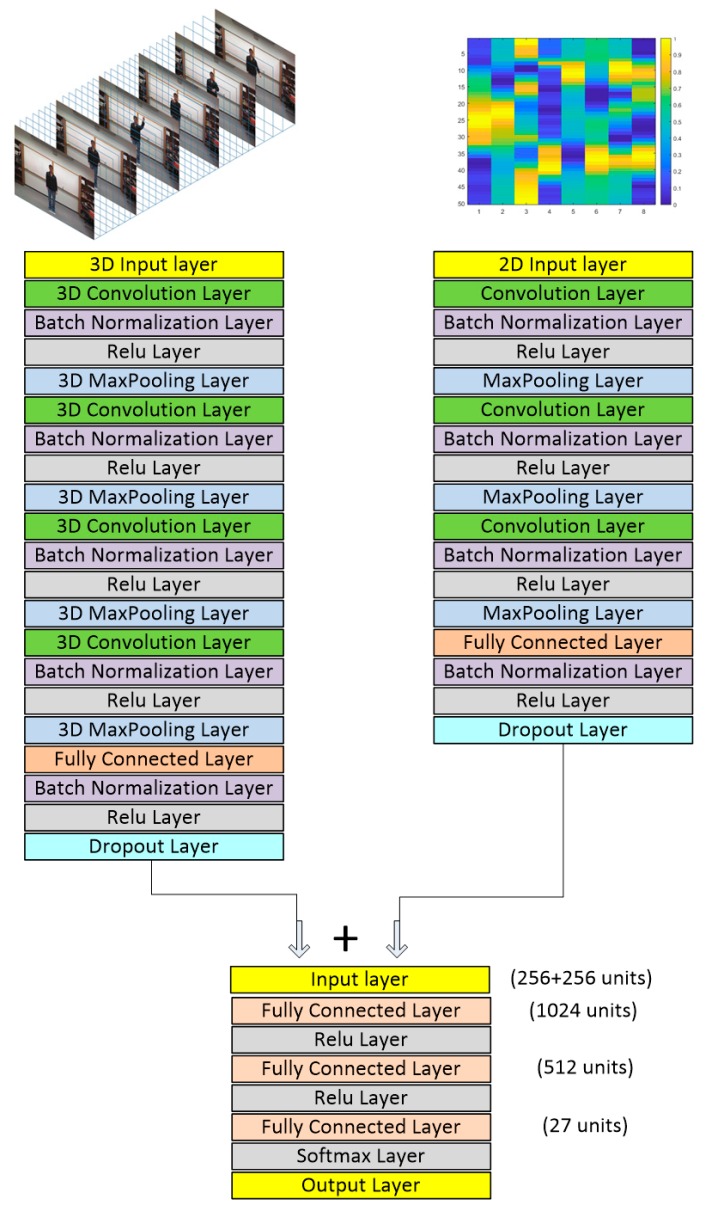
Network architecture used for the feature-level fusion of video and inertial sensing modalities.

Besides the above decision-level fusion approach of combining the decisions of two deep neural networks, one for video and one for inertial sensing, a feature-level fusion approach is also considered in this work. 256 features are extracted from the video network model and the inertial network model dropout layers as indicated in Figure 4. Then, these features are concatenated to form a 512-dimensional vector. This vector is used as the input to a backpropagation neural network with three fully connected layers based on the ReLU activation function and the softmax cost function. The final output of the last layer is used as the score of the classes corresponding to the 27 actions. The class or action with the highest score is taken to be the recognized action.

## 4. Experimental Results 

**Figure 5:**
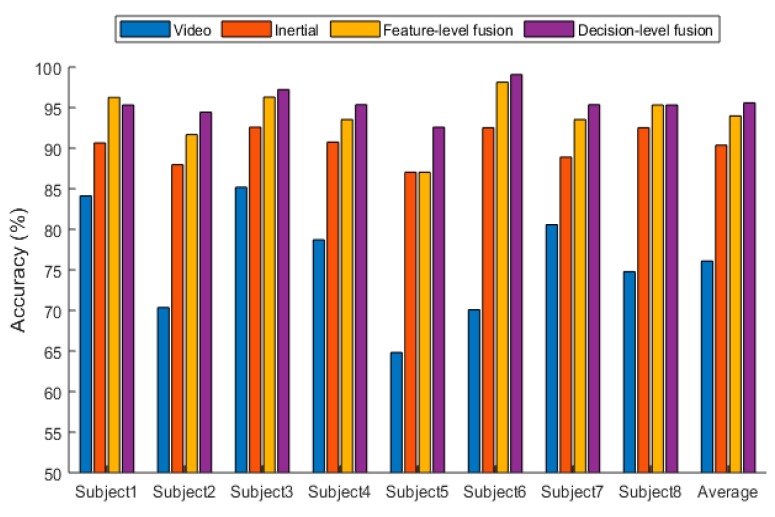
Recognition accuracy for the four situations of sensing modality across the eight subjects in UTD-MHAD dataset.

This section presents the results of the experiments conducted to examine the effectiveness of the introduced fusion approach when the two sensing modalities of video images and inertial signals are used simultaneously. A leave-one-out cross validation was performed, meaning that the data from one subject was used for testing, and the data from the remaining seven subjects were used for training. This process was repeated for each of the subjects. Then, an average was taken across the subjects. The average accuracy of the following four approaches were obtained: (1) only using video sensing modality, (2) only using inertial sensing modality, (3) feature-level fusion of video and inertial sensing modalities, and (4) decision-level fusion of video and inertial sensing modalities. Table 4 provides the average recognition accuracy obtained for these four situations on the UTD-MHAD dataset, and Figure 5 illustrates the recognition performance for each of the eight subjects in the UTD-MHAD dataset.

**Figure 6:**
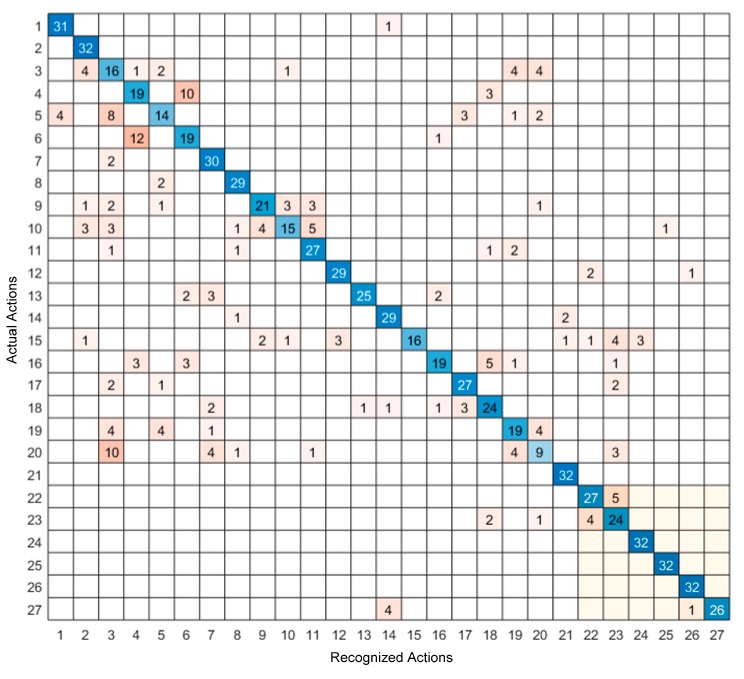
Confusion matrix of the video only sensing modality.

**Figure 7:**
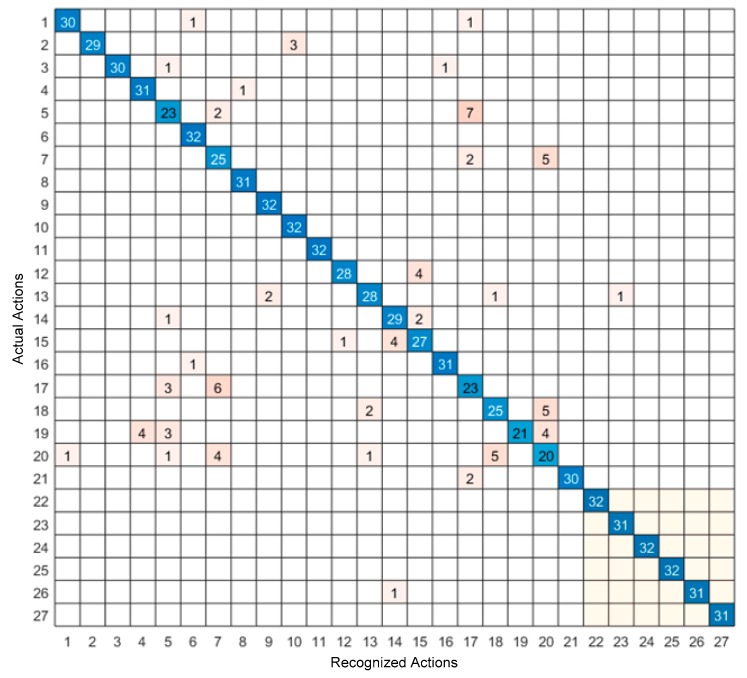
Confusion matrix of the inertial only sensing modality.

**Figure 8:**
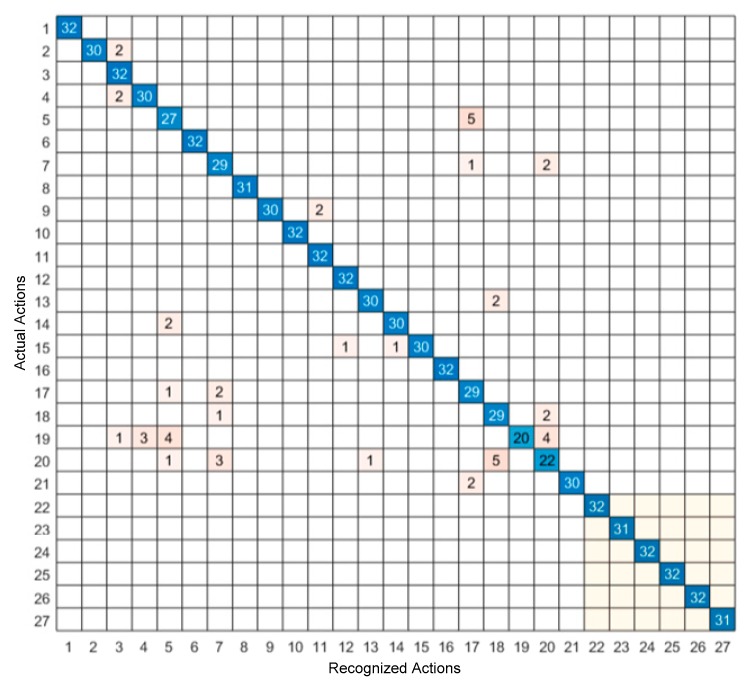
Confusion matrix of the feature-level fusion of video and inertial sensing modalities.

**Figure 9:**
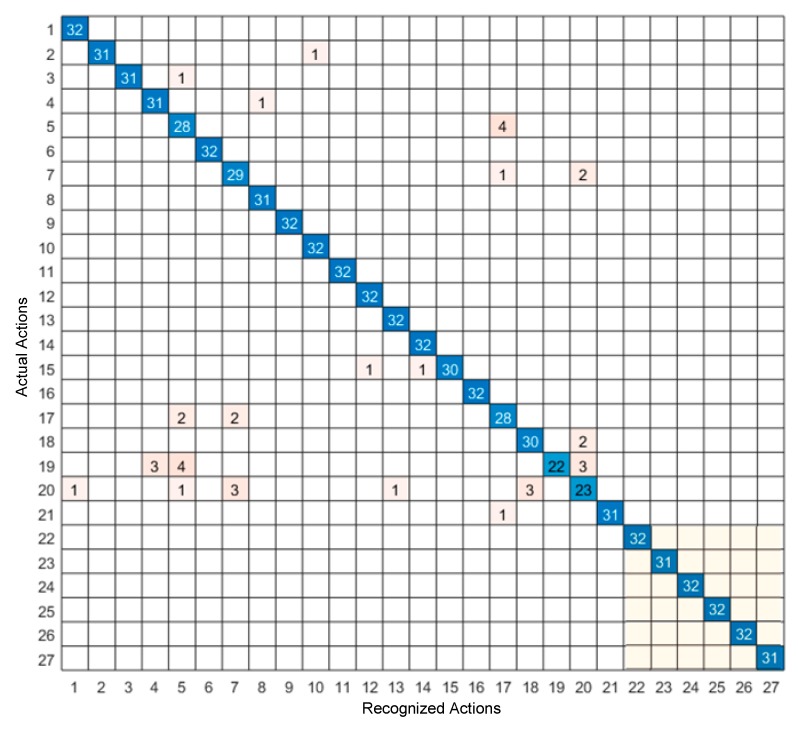
Confusion matrix of the decision-level fusion of video and inertial sensing modalities.

The confusion matrix of the video sensing modality only and the inertial sensing modality only are shown in Figure 6 and Figure 7, respectively, and the confusion matrix of the feature-level fusion modality and the decision-level fusion modality are shown in Figure 8 and Figure 9, respectively. The bottom gray parts of these confusion matrices correspond to leg actions (actions 22 through 27). 

## 5. Discussion of Results

From Table 4, it can be seen that both the feature-level fusion and decision-level fusion generated higher accuracies than the individual sensing modalities. In addition, the decision-level fusion generated fewer errors compared to the feature-level fusion. More specifically, as can be seen from Figure 6, most of the errors occurred between actions 4 and 6, between actions 3 and 20, between actions 3 and 5, and between actions 22 and 23. These errors were caused due to the similarities of the volume data between these actions. For example, the images associated with action 4 corresponding to two hand clap, and action 6 corresponding to crossing arms appeared close. From Figure 7, it can be seen that most of the errors occurred between actions 18 and 20, between actions 5 and 17, between actions 7 and 20, and between actions 7 and 17. These errors were caused by the similarities of the inertial signals between these actions. For example, the inertial signals associated with action 7 corresponding to shooting a basketball, action 17 corresponding to a tennis serve, and action 20 corresponding to a right-hand catch appeared close. As can be seen from Figure 8 and Figure 9, in both the feature-level fusion and decision-level fusion, most of the errors occurred between actions 18 and 20 and actions 5 and 17, with fewer number of errors compared to the single sensing modalities, indicating the positive impact made by fusing the individual sensing modalities. From the experiments conducted, both the feature-level fusion and the decision-level fusion exhibited more robustness to errors compared to individual sensing modalities.

## 6. Conclusions

In this paper, for the first time, the simultaneous utilization of video and inertial sensing modalities were considered within a fusion framework to achieve human action recognition based on deep learning models. The following four approaches were compared in terms of recognition accuracies: using only video data as input to a deep neural network, using only inertial data as input to a deep neural network, using both video data and inertial data as inputs to two deep neural networks within a decision-level fusion framework, and using both video data and inertial data as inputs to two deep neural networks within a feature-level fusion framework. The experiments conducted based on the publicly available UTD-MHAD dataset have shown that the decision-level fusion approach provided the highest recognition accuracy of 95.6%—The fusion of the two sensing modalities exhibited more robust recognition outcome in terms of misclassification errors compared to the situations when each sensing modality was used individually. 

## Figures and Tables

**Table 1:** Actions in the University of Texas at Dallas Multimodal Human Action Dataset (UTD-MHAD) dataset.

Action Number	Hands Actions	Action Number	Legs Actions
1	right arm swipe to the left	22	jogging in place
2	right arm swipe to the right	23	walking in place
3	right hand wave	24	sit to stand
4	two hand front clap	25	stand to sit
5	right arm throw	26	forward lunge (left foot forward)
6	cross arms in the chest	27	squat (two arms stretch out)
7	basketball shoot		
8	right hand draw x		
9	right hand draw circle (clockwise)		
10	right hand draw circle (counter clockwise)		
11	draw triangle		
12	bowling (right hand)		
13	front boxing		
14	baseball swing from right		
15	tennis right hand forehand swing		
16	arm curl (two arms)		
17	tennis serve		
18	two hand push		
19	right hand knock on door		
20	right hand catch an object		
21	right hand pick up and throw		

**Table 2:** Architecture of the 3D convolutional neural network.

Layers and Training Parameters	Values
Input layer	320 × 240 × 32
1st 3D convolutional layer	16 filters, filter size 3 × 3 × 3, stride 1 × 1 × 1
2nd 3D convolutional layer	32 filters, filter size 3 × 3 × 3, stride 1 × 1 × 1
3rd 3D convolutional layer	64 filters, filter size 3 × 3 × 3, stride 1 × 1 × 1
4th 3D convolutional layer	128 filters, filter size 3 × 3 × 3, stride 1 × 1 × 1
All 3D max pooling layer	pooling size 2 × 2 × 2, stride 2 × 2 × 2
1st fully connected layer	256 units
2nd fully connected layer	27 units
dropout layer	50%
Initial learn rate	0.0016
Learning rate drop factor	0.5
Learn rate drop period	4
Max epochs	20

**Table 3:** Architecture and training parameters of the 2D convolutional neural network.

Layers and Training Parameters	Values
Input layer	8 × 50
1st 2D convolutional layer	16 filters, filter size 3 × 3, stride 1 × 1
2nd 2D convolutional layer	32 filters, filter size 3 × 3, stride 1 × 1
3rd 2D convolutional layer	64 filters, filter size 3 × 3, stride 1 × 1
All 2D max pooling layer	pooling size 2 × 2, stride 2 × 2
1st fully connected layer	256 units
2nd fully connected layer	27 units
dropout layer	50%
Initial learn rate	0.0016
Learning rate drop factor	0.5
Learn rate drop period	4
Max epochs	20

**Table 4:** Average accuracy of video sensing modality only, inertial sensing modality only, feature-level fusion of video and inertial sensing modalities, and decision-level fusion of video and inertial sensing modalities.

Approaches	Average Accuracy (%)
Video only	76.0
Inertial only	90.3
Feature-level fusion of video and inertial	94.1
Decision-level fusion of video and inertial	95.6
